# Awareness, Knowledge, and Attitudes Toward Venous Thromboembolism (VTE) Among the Population of Al-Baha Region, Saudi Arabia

**DOI:** 10.7759/cureus.99795

**Published:** 2025-12-21

**Authors:** Omar Ballut, Ali M Alqarni, Muhannad Ahmad Alzahrani, Omran M Alzahrani, Rashed Alghamdi, Anas Alalyani, Talal Alzahrani, Mohammed Ali Alzahrani, Saeed Saleh Aziz Alghamdi, Hesham Alharbi, Wejdan Alzahrani, Saad Alghamdi, Mubarak Aldosari

**Affiliations:** 1 Internal Medicine, King Fahad Hospital, Albaha, SAU

**Keywords:** al-baha, attitude, awareness, knowledge, ksa, venous thromboembolism (vte)

## Abstract

Introduction: Venous thromboembolism (VTE) is a serious and potentially life-threatening condition, yet public understanding of its risk factors, symptoms, and prevention remains limited in many regions of Saudi Arabia. This study aimed to assess awareness, knowledge, and attitudes toward VTE among the general population in the Al-Baha region.

Methods: A descriptive cross-sectional study was conducted among adults in Al-Baha using a self-administered questionnaire distributed physically and electronically. Convenience sampling was utilized. Data were analyzed using SPSS Version 27 (IBM Corp., Armonk, NY, USA). Variables associated with VTE knowledge in bivariate analysis (p < 0.25) were included in a multivariate logistic regression model to determine independent predictors.

Results: Among 387 participants, only 149 (38.5%) demonstrated good knowledge of VTE. Bivariate analysis showed significant associations between knowledge level and age, sex, personal history of venous thrombosis, and knowing someone with venous thrombosis (all p < 0.05). However, in the multivariate model, only personal history of venous thrombosis (AOR = 4.16; 95% CI: 1.302-13.278), knowing someone with VTE (AOR = 2.86; 95% CI: 1.787-4.590), and sex (AOR = 0.58; 95% CI: 0.351-0.949) remained significant predictors. Notable knowledge gaps were observed regarding oral contraceptive-related VTE risk and postoperative thromboprophylaxis, particularly among recently hospitalized participants.

Conclusion: Public knowledge of VTE in the Al-Baha region was generally inadequate. Personal experience with VTE and female sex were the strongest predictors of better knowledge. Substantial misconceptions and knowledge deficits - especially concerning oral contraceptive pill-related risk and awareness of hospital-provided preventive measures - underscore the need for targeted educational interventions to improve VTE prevention and early recognition.

## Introduction

Venous thromboembolism (VTE) remains a major health challenge, affecting about 10 million patients globally every year [1]. It is the third most common cause of cardiovascular death after heart attack and stroke [2], highlighting the level of health risk it poses. VTE comprises of deep vein thrombosis (DVT), which refers to the formation of a blood clot in a deep vein, mostly in the legs, and pulmonary embolism (PE), which is a condition that arises if/when a part of the clot goes to the lungs and obstructs blood flow, potentially causing death [3]. Some of the common risk factors of VTE include old age, obesity, smoking, history of DVT, pregnancy, surgery, and hospitalizations [4,5]. Additionally, asymptomatic VTE is common and can often go undetected, while most symptoms of DVT or PE are similar to the symptoms of other conditions, creating a high possibility of misdiagnosis [6]. Because many VTE cases present with subtle or nonspecific symptoms, they are often mistaken for less serious conditions. This overlap increases the likelihood of delayed recognition within the community and makes accurate symptom identification more challenging for the public.

In Saudi Arabia, certain factors increase the risk of exposure to VTE. These include, but are not limited to, relatively high obesity and metabolic syndrome rates, a big number of people living sedentary lifestyles, and high exposure to hereditary clotting disorders [7]. This makes it important that populations in different parts of the country have adequate understanding of the condition for them to be sufficiently equipped to protect themselves. However, a study conducted in Riyadh revealed that only about 18.6% of adult residents were aware of DVT [8]. Such low numbers indicate that not only are many people exposed to the risk of the condition but are also unaware of that exposure. In 2014, the International Society on Thrombosis and Haemostasis (ISTH) declared 13 October as World Thrombosis Day as a strategy to increase awareness and knowledge of the condition among the global general population [9].

In line with the campaign by the ISTH, this study seeks to assess the awareness, knowledge, and attitudes toward VTE among the general population in Al-Baha, Saudi Arabia. This study will not only assess public awareness of VTE but also provide critical insights regarding the differences that may exist in such awareness levels from one region to another. Results of the study will contribute significantly to the formulation of healthcare policy and planning, providing the needed data and scientific evidence for targeted educational campaigns in Al-Baha. Therefore, this study will contribute to the creation of increased awareness and prompt healthier behaviors among at-risk individuals.

## Materials and methods

Study design and setting

This study utilized a descriptive, cross-sectional design to evaluate the awareness, knowledge, and attitudes toward VTE among the general population in the Al-Baha region of Saudi Arabia. Data collection was conducted across various public places within Al-Baha, including markets, parks, and social institutions, as well as through online social media platforms to ensure broad participation and representation. The study was carried out from 23 July 2025 to 23 October 2025.

Study population and sampling

The study population consisted of adults aged 18 years and above residing in the Al-Baha region. A convenience sampling approach was used, whereby eligible residents who met the inclusion criteria were invited to participate until the desired sample size was reached. Individuals were included if they were Saudi or non-Saudi residents living in Al-Baha for at least six months, aged 18 years or older, and willing to participate voluntarily by providing informed consent. Participants were excluded if they were healthcare professionals, to avoid potential bias due to professional medical knowledge, or if they had cognitive limitations that could hinder their ability to understand the questionnaire.

Sample size calculation

The minimum required sample size was determined using the Raosoft sample size calculator, applying a 95% confidence level, a 5% margin of error, and an estimated response distribution of 50% to ensure adequate statistical power. This calculation yielded a minimum required sample size of 385 participants. A total of 387 individuals completed the survey, meeting the expected sample threshold.

Data collection tool and technique

Data were collected using a structured questionnaire distributed physically and electronically. The questionnaire was developed by a clinical specialist (consultant) with expertise in venous thromboembolism and public health, ensuring that all items were relevant and aligned with established VTE knowledge domains. A pilot test was conducted among 15 participants to assess clarity, comprehension, and content suitability, and the pilot test yielded high clarity, relevance, and acceptability, confirming that participants were able to understand and respond to the questionnaire items appropriately. Data were collected using a validated structured questionnaire distributed physically and electronically. The survey was self-administered and consisted of four main sections: (1) sociodemographic characteristics, (2) knowledge regarding VTE including risk factors, symptoms, prevention, and complications, (3) awareness and general familiarity with VTE, and (4) attitudes toward VTE prevention, symptom response, and behavior. Knowledge scores were categorized as “good” or “poor” based on predefined scoring criteria, where correct responses were summed and compared against a calculated threshold. Knowledge scoring was based on participants’ responses to knowledge-related items. All correct answers were assigned a score of 1, while incorrect answers were assigned a score of 0. Participants who achieved 75% or more of the total possible score were categorized as having good knowledge, whereas those scoring below this threshold were classified as having poor knowledge [10].

Statistical analysis

Data were coded, entered, and analyzed using SPSS Version 27 (IBM Corp., Armonk, NY, USA). Descriptive statistics were used to summarize variables, with frequencies and percentages reported for categorical variables and mean ± standard deviation for continuous variables. The level of knowledge was categorized into good and poor knowledge groups based on scoring distribution. The Chi-square test was applied to assess associations between knowledge level and demographic characteristics. Variables with a significance value of p < 0.25 in the bivariate analysis were included in a multivariate logistic regression model to identify independent predictors of good VTE knowledge. Results were expressed as Adjusted Odds Ratios (AOR) with corresponding 95% Confidence Intervals (CI). A p-value of < 0.05 was considered statistically significant.

Ethical considerations

Ethical approval for the study was obtained from the institutional ethical committee prior to initiation of data collection. Participation was entirely voluntary, and written informed consent was obtained from each participant. No personal identifiers were collected, and the confidentiality and anonymity of all respondents were strictly maintained. The study adhered to ethical standards outlined in the Declaration of Helsinki, and participants were assured that their responses would be used solely for research purposes.

## Results

Table 1 shows that a total of 387 participants were included in the study. A majority of the participants (275, 71.1%) were males; with more than half (261, 67.4%) with university education. Furthermore, the participants were distributed nearly evenly across the age groups.

**Table 1:** Demographic data presented in frequencies (n) and proportion (%)

Demographic data	Category	Frequency (%)
Age	18-26	86 (22.2%)
	27-35	74 (19.1%)
	36-45	80 (20.7%)
	46-55	92 (23.8%)
	>55	55 (14.2%)
Gender	Female	112 (28.9%)
	Male	275 (71.1%)
Education level	Secondary	94 (24.3%)
	University	261 (67.4%)
	Other	32 (8.3%)

Knowledge about risk factors, symptoms, prevention and complications (Table 2) revealed a considerable proportion of the participants had heard of venous thromboembolism (234, 60.5%), deep vein thrombosis (232, 59.9%) and pulmonary embolism (268, 69.3%). Nearly half (202, 52.2%) had received medical education information about stroke. Notably, only 17 participants (4.4%) reported a history of venous thrombosis while a majority (238, 61.5%) knew someone with venous thrombosis 

**Table 2:** Knowledge about risk factors, symptoms, prevention and complications Knowledge about risk factors, symptoms, prevention and complications presented in frequencies (n) and proportion (%)

Questions	Category	Frequency (%)
Have you ever heard of venous thromboembolism (VTE)?	No	153 (39.5%)
	Yes	234 (60.5%)
Have you ever heard of a leg clot (deep vein thrombosis-DVT)?	No	155 (40.1%)
	Yes	232 (59.9%)
Have you heard of a pulmonary embolism (PE)?	No	119 (30.7%)
	Yes	268 (69.3%)
Have you ever received medical or educational information about strokes?	No	185 (47.8%)
	Yes	202 (52.2%)
Have you ever had a venous thrombosis?	No	370 (95.6%)
	Yes	17 (4.4%)
Do you know anyone who has had a venous thrombosis?	No	149 (38.5%)
	Yes	238 (61.5%)

**Figure 1:**
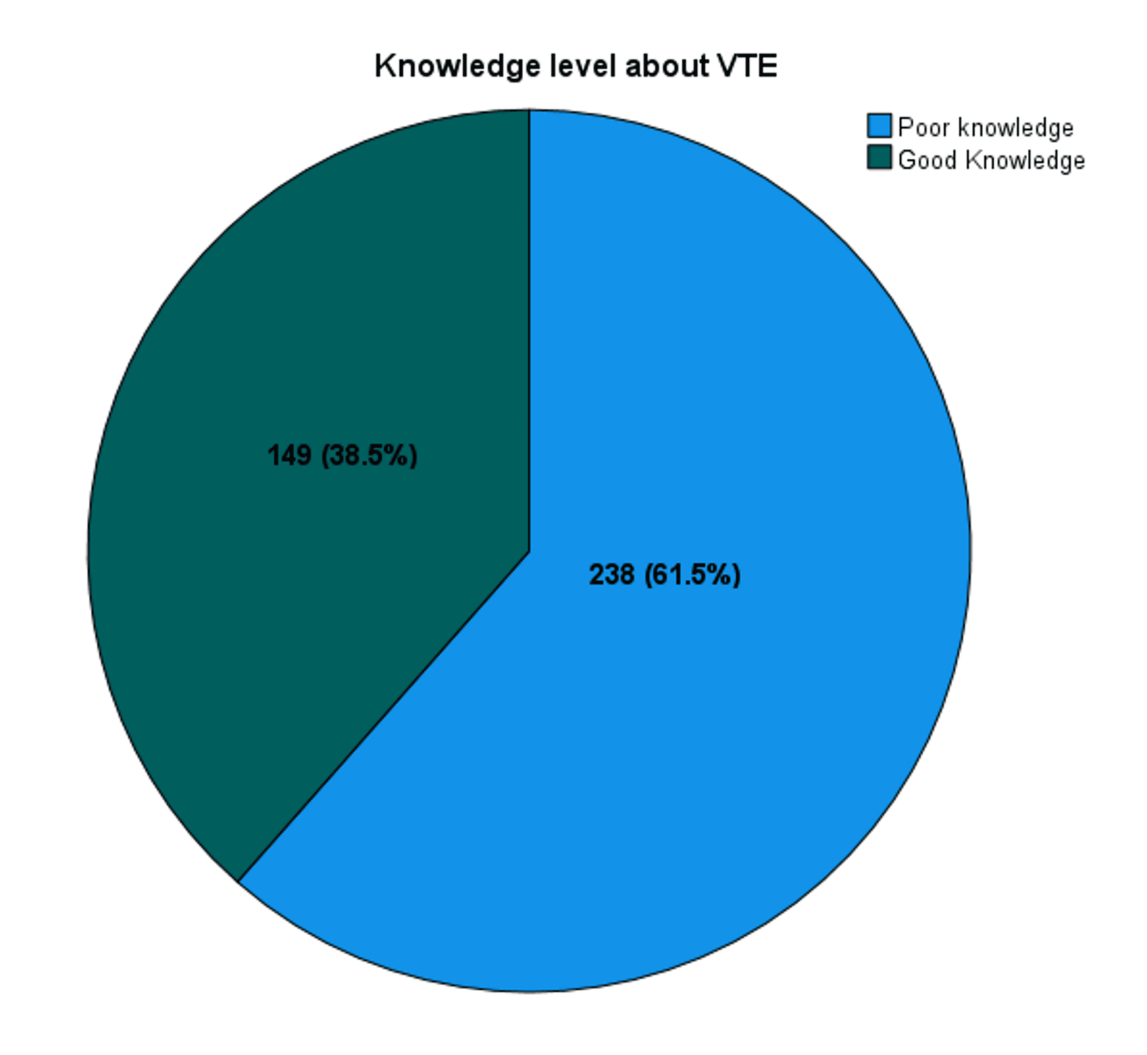
Knowledge level about venous thromboembolism (VTE).

Figure 1 illustrates participants’ knowledge about risk factors, symptoms, prevention and complications of VTE. About 149 participants (38.5%) had good knowledge while a majority, 238, of participants (61.5%) had poor knowledge about VTE.

Awareness and general familiarity with VTE (Table 3) revealed that an overwhelming majority (366, 94.6%) were aware that physical activity helps in reducing the risk of venous thromboembolism, with only 43 participants (11.1%) believing that doctors do not explain to patients the risk of venous thromboembolism after surgery. The common risk factors for venous thromboembolism reported were obesity (295, 76.2%) and smoking (260, 67.2%). The frequently mentioned symptoms of the DVT were leg pain (278, 71.8%) and swelling (253, 65.4%) and the common symptoms of pulmonary embolism reported were shortness of breath (315, 81.4%) and chest pain (247, 63.8%). However, only 140 participants (36.2%) were aware that oral contraceptive pills (OCPs) increase the risk of venous thromboembolism

**Table 3:** Awareness (general familiarity with venous thromboembolism (VTE)) Awareness and general familiarity with VTE presented in frequencies (n) and proportion (%)

Questions	Category	Frequency (%)
Do you think physical activity helps reduce the risk of venous thromboembolism (VTE)?	No	21 (5.4%)
	Yes	366 (94.6%)
Do you think doctors usually explain to patients the risk of venous thromboembolism after surgery?	Maybe	171 (42.2%)
	No	43 (11.1%)
	Yes	173 (44.7%)
What are the risk factors for venous thromboembolism?	Surgical procedures	165 (42.6%)
	Lack of movement	167 (43.2%)
	Pregnancy	86 (22.2%)
	Cancer	101 (26.1%)
	Smoking	260 (67.2%)
	Obesity	295 (76.2%)
	Long travel	102 (26.4%)
	Lack of exercise	153 (39.5%)
	Blood diseases	195 (50.4%)
What are the common symptoms of deep vein thrombosis (DVT)?	Leg pain	278 (71.8%)
	Swelling	253 (65.4%)
	Redness	131 (33.9%)
	Warmth in the affected limb	119 (30.8%)
What are the symptoms of pulmonary embolism (PE)?	Shortness of breath	315 (81.4%)
	Chest pain	247 (63.8%)
	Tachycardia	9 (2.3%)
	Cough with blood	159 (41.1%)
	Rapid heartbeat	130 (33.6%)
Can oral contraceptive pills increase the risk of venous thromboembolism?	Yes	140 (36.2%)
	No	22 (5.7%)
	Maybe	225 (58.1%)

Participants’ attitude towards symptoms and preventive behavior (Table 4) revealed that nearly three-quarters (292, 75.5%) believed that VTE can be prevented. Additionally, A majority (286, 73.9%) expressed their willingness to seek medical attention in case of swelling or pain in their legs. An overwhelming majority (364, 94.1%) acknowledged the importance of venous thromboembolism awareness campaigns. Furthermore, a substantial proportion of patients hospitalized were not aware of the medication to prevent venous thromboembolism and the risk of developing venous thromboembolism (19, 44.2%).

**Table 4:** Attitudes (reaction to symptoms, preventive behavior) Attitude (reaction to symptoms, preventive behaviour) presented in frequencies (n) and proportion (%)

Statement	Category	N (%)
Have you ever heard of anticoagulant medications?	No	113 (29.2%)
	Yes	274 (70.8%)
Do you think venous thromboembolism can be prevented?	Maybe	90 (23.3%)
	No	5 (1.3%)
	Yes	292 (75.5%)
If you felt swelling or pain in your leg, would you seek medical help?	Maybe	77 (19.9%)
	No	24 (6.2%)
	Yes	286 (73.9%)
Do you think venous thromboembolism campaigns are important?	Maybe	16 (4.1%)
	No	7 (1.8%)
	Yes	364 (94.1%)
Do you think young people are also prone to venous thromboembolism?	Maybe	76 (19.6%)
	No	12 (3.1%)
	Yes	299 (77.3%)
Have you been hospitalized within the last 12 months?	No	344 (88.9%)
	Yes	43 (11.1%)
If yes, have you been advised to move to avoid venous thromboembolism?	No	7 (16.3%)
	Yes	36 (83.7%)
Were you given medications to prevent clots?	I don’t know	5 (11.6%)
	No	15 (34.9%)
	Yes	23 (53.5%)
After leaving the hospital, were you alerted to the risk of venous thromboembolism?	No	19 (44.2%)
	Yes	24 (55.8%)
Do you think surgery patients or long-term patients are at risk for venous thromboembolism?	Maybe	109 (28.2%)
	No	17 (4.4%)
	Yes	261 (67.4%)
Have you heard of compression stockings or other protective devices used in hospitals?	No	201 (51.9%)
	Yes	186 (48.1%)

Participants’ attitude towards symptoms and preventive behavior (Table 4) revealed that nearly three-quarters (292, 75.5%) believed that VTE can be prevented. Additionally, a majority (286, 73.9%) expressed their willingness to seek medical attention in case of swelling or pain in their legs while a notable proportion of them (19.9%) were uncertain (maybe) and about 6.2% of them would not seek help. An overwhelming majority (364, 94.1%) acknowledged the importance of venous thromboembolism awareness campaigns. Furthermore, a substantial proportion of hospitalized patients (19, 44.2%) were not alerted to the risk of VTE upon leaving the hospital, and about 15 (34.9%) were not given medication to prevent clots.

The results (Table 5) revealed statistically significant associations between participants' age, gender, history of venous thrombosis, knowing someone with venous thrombosis and knowledge about VTE (p <0.001, p=0.023, p=0.001, p=<0.001). A significant proportion of participants aged below 35 years, females, those with history of venous thrombosis and those who knew someone with venous thrombosis demonstrated good knowledge about VTE. However, no significant associations were observed between education level and knowledge about VTE (p > 0.05).

**Table 5:** The association between demographic information and knowledge about venous thromboembolism (VTE) * Significant at p<0.05 level.

Variables	Knowledge about VTE				
	Category	Poor	Good	Pearson 𝜒2 value	p-value
Age	18-26	55 (64.0%)	31 (36.0%)	20.867	<0.001*
	27-35	29 (39.2%)	45 (60.8%)		
	36-45	58 (72.5%)	22 (27.5%)		
	46-55	61 (66.3%)	31 (33.7%)		
	>55	35 (63.6%)	20 (36.4%)		
Gender	Female	59 (52.7%)	53 (47.3%)	5.179	0.023*
	Male	179 (65.1%)	96 (34.9%)		
Education level	Secondary	60 (63.8%)	34 (36.2%)	2.038	0.361
	University	162 (62.1%)	99 (37.9%)		
	Other	16 (50.0%)	16 (50.0%)		
History of venous thrombosis	No	234 (63.2%)	136 (36.8%)	10.826	0.001*
	Yes	4 (23.5%)	13 (76.5%)		
Knowing someone with venous thrombosis	No	114 (76.5%)	35 (23.5%)	23.058	<0.001*
	Yes	124 (52.1%)	114 (47.9%)		

In the multivariate logistic regression model (Table 6), history of venous thrombosis emerged as the most significant predictor of good knowledge about VTE (AOR=4.16; 95% CI = 1.302- 13.278; p=0.016), followed by knowing someone with venous thrombosis (AOR=2.86; 95% CI = 1.787- 4.590; p<0.001) and sex (AOR=0.58; 95% CI = 0.351- 0.949; p=0.030). Other factors were not statistically significant.

**Table 6:** Multivariate logistic regression analysis of factors associated with the good knowledge about venous thromboembolism (VTE) AOR – Adjusted Odds Ratio; CI – Confidence Interval ** Significant at p<0.05 level

Variables	AOR	95% CI	P-value
Age	0.94	0.795– 1.116	0.490
Gender	0.58	0.351– 0.949	0.030**
Education level	1.17	0.784– 1.751	0.441
History of venous thrombosis	4.16	1.302– 13.278	0.016**
Knowing someone with venous thrombosis	2.86	1.787– 4.590	<0.001**

## Discussion

Venous thromboembolism is a global public health concern which has been linked to sudden death and serious long-term health effects if not treated quickly and effectively. Despite these adverse health effects, there is limited research on the awareness of VTE among the general population in Saudi Arabia [10,11]. This study aimed to bridge this gap by assessing the awareness, knowledge and attitude towards VTE among the general population of Al-Baha region, Saudi Arabia.

The study found inadequate knowledge about VTE among the general population of Al-Baha, with a majority (238, 61.5%) of them demonstrating poor knowledge about the condition. The finding is consistent with the Saudi study conducted by Elmahdi et al., which reported a deficit in knowledge and awareness about VTE, particularly among the male population [12].

Personal history of venous thrombosis was the most significant predictor of good knowledge about VTE (AOR=4.16; 95% CI = 1.302- 13.278; p=0.016), with a notable proportion of participants who had experienced venous thrombosis demonstrating good knowledge about VTE than those who did have the experience (p=0.001). The finding aligns with those of a Jordan study conducted by Jarab et al., who reported prior VTE as a stronger predictor of awareness, with participants without personal history of VTE having much higher odds of being unaware compared to those who had prior thrombosis [13]. Similarly, the study conducted by Okoye et al. reported strong correlation between the personal experience with thrombosis and VTE knowledge [14].

The study observed that females had considerably better knowledge about VTE than males (p=0.023). The finding concurs with those of a Saudi study by Alyahya et al., who noted that females had significantly higher awareness of pulmonary embolism and deep vein thrombosis compared to their male counterparts [15]. The poor awareness among males highlights the need for health education enhancements with key interest in raising awareness levels among the male gender as well.

Although age was not a significant predictor of good knowledge based on multivariate results, the study observed significant age difference in knowledge levels, with a considerable proportion of participants aged 35 years and below showing good knowledge of VTE. The finding is consistent with those of a Saudi study by Alaklabi et al., who found the awareness of VTE varying significantly with age [8]. This observation could be attributed to the facts that individuals in this age group are mostly in institutions of higher learning with exposure to health information through reliable medical sources, internet and social media.

Knowing someone with venous thrombosis was also found to be a significant predictor of good knowledge (AOR=2.86; 95% CI = 1.787- 4.590; p<0.001), with a considerable proportion of those who knew someone with thrombosis showing significantly better knowledge of VTE than those who did not (p<0.001). This observation could be likely due to shared experiences and personal connections and attention which ultimately increase the awareness of the condition.

The study revealed that the commonly recognized risk factors for stroke were obesity (295, 76.2%) and smoking (260, 67.2%); consistent with the Saudi study by Maqbul et al. which reported smoking as the most commonly recognized risk factor of VTE [16]. Additionally, the frequently mentioned symptoms of a leg clot were leg pain (278, 71.8%) while the most commonly recognized symptom of pulmonary embolism was shortness of breath (315, 81.4%).

The study found significant gaps in knowledge regarding the risks associated with OCPs, with only 140 participants (36.2%) aware that OCPs increase VTE risk. The findings emphasize the need for targeted education about OCPs and VTE risk especially for younger women, women of reproductive age and healthcare prescribers.

The study noted significant care gaps, with a considerable proportion of hospitalized patients (19, 44.2%) not alerted to the risk of VTE upon leaving the hospital, and about 15 patients (34.9%) not given medication to prevent clots. The study findings are similar to those of Bosaeed et al. who found that hospitalized patients received insufficient medical advice regarding the risk of clot and their preventive measures [17]. These findings highlight institutional care gaps and serious failure on the part of the hospital, underscoring the urgent need for policies that improve medication counseling, promote proper medication use and prevent potential risk and side effects.

The study revealed positive attitude towards VTE prevention, with nearly three-quarters of the participants (286, 73.9%) expressing their willingness to seek medical attention upon noticing VTE symptoms. Furthermore, the overwhelming majority (364; 94.1%) recognized the importance of stroke awareness campaigns, highlighting strong support for public education on VTE.

A number of limitations and shortcomings were considered while evaluating the results. The cross-sectional design could only assess the relationship between the study attributes but not their causalities. Due to the reliance on convenience sampling, the results may not accurately reflect the broader population of Al-Baha. It is possible that individuals who were more readily available, such as regular visitors to public spaces or those active on social media, are overrepresented in the sample. This method of sampling could have led to selection bias and restricted the applicability of the findings. Furthermore, since the study relied on self-reported questionnaire data, biases related to recollection and social desirability may have compromised its reliability and accuracy. The conclusions of the study cannot be generalized to the other population, given that it was conducted in only one region, Al-Baha.

## Conclusions

The study found inadequate knowledge about venous thromboembolism among the general population of Al-Baha, Saudi Arabia. Good knowledge of VTE was associated with personal history of venous thrombosis and female gender. The study noted significant knowledge gaps regarding the risks of clots and the medication used to prevent them, particularly among participants who had been hospitalized, highlighting the need for targeted educational interventions to increase awareness, improve prevention and management of VTE, and ultimately reduce its burden on public health.
